# Effect of PMMA Coupling Layer in Enhancing the Ultrasonic Weld Strength of Novel Room Temperature Curable Acrylic Thermoplastic to Epoxy Based Composites

**DOI:** 10.3390/polym14091862

**Published:** 2022-05-02

**Authors:** Goram Gohel, Chun Zhi Soh, Kah Fai Leong, Pierre Gerard, Somen K. Bhudolia

**Affiliations:** 1 School of Mechanical and Aerospace Engineering, Nanyang Technological University, 50 Nanyang Avenue, Singapore 639798, Singapore; sohc0019@e.ntu.edu.sg (C.Z.S.); mkfleong@ntu.edu.sg (K.F.L.); 2 Groupement de Recherche de Lacq, Arkema, Route Départementale 817, BP 34, 64170 Lacq, France; pierre.gerard@arkema.com

**Keywords:** ultrasonic welding, thermoplastic, thermoset, lap shear strength, interphase

## Abstract

The joining of composites can be performed in an extremely short time with more energy-efficient ultrasonic welding techniques. The current research investigated the performance optimization of ultrasonic welding of carbon/Elium^®^ composite to carbon/epoxy composite using a polymethyl methacrylate (PMMA) coupling interlayer. The weld strength was quantified by static lap shear strength (LSS) testing. A new methodology was used by creating a PMMA coupling layer on the epoxy composite adherend to achieve an improved interphase and thus enhance the weld properties. The LSS of Elium (EL)-Epoxy (EP) _0.25_0.25 was found to be 190% higher compared to that of EL-EP, confirming the effectiveness of the strategy used for creating an interlayer thermoplastic coupling layer. The time required for welding was optimized to be 2s as compared to 10 min required for adhesive bonding. Scanning electron microscopic images of epoxy and PMMA/Elium matrix interphase were observed to have a rough surface and remained largely unaffected by welding. There was an interphase change further away from the interphase to a rougher texture. There was little to no effect on the penultimate layer on the weld strength, as no interphase change could be observed after welding. Fractography investigation revealed shear cusps, matrix plastic deformation, fiber imprints, fiber pull-out, and good adhesion between matrix and fiber, features seen for configuration with maximum LSS. The current research findings present a way to join Elium^®^ with epoxy composites that could be used in applications that require a selective strengthening, such as in sporting goods and consumer products. Furthermore, a detailed investigation is ongoing to use different filler particles and coupling layers to reach the maximum welding performance.

## 1. Introduction

Demand for thermoplastic composites has increased due to the factors of lower cost, higher productivity, better facture toughness [1,2], vibration damping [3,4], recyclability [5], and weldability [6,7,8] as compared to thermosets. The joining of composite parts can account for a significantly large percentage of the total manufacturing cost because conventional methods, such as mechanical fastening and adhesives, involve many steps that are labor-intensive and time-consuming. Mechanical fastening, such as with bolts and nuts, increases the total weight of the component and causes cracks due to the stress concentration of drilled holes. On the other hand, adhesives require extensive surface preparation and time for curing [9,10,11]. The most common type of fusion welding techniques for thermoplastic composites are induction [12,13,14], resistance, [15,16,17] and ultrasonic welding [13,18,19,20,21,22,23]. Ultrasonic welding (UW) is more suitable for thermoplastic, as it can produce higher weld strength, requires an extremely short time, and can be used for automation [24].

Many industries have transitioned from conventional metal materials to composite materials due to properties such as light weight, higher stiffness, higher strength, etc. A technological shift in the manufacturing process is required, as the cost of manufacturing composite parts is too high. To reduce the cost of the final part, the key focus is on the material selection, manufacturing process, and finishing steps. Some of the areas of research as mentioned in an industrial report [25] for the technological improvement of composite manufacturing are reductions in the cost of raw material, automated mass production, bonding methods, recyclability, and the repair of composite parts. Manufacturing large composite parts as a single part is complex and hence, they are commonly manufactured using many small components and bonded together using adhesive [26,27]. Adhesive bonding, fusion bonding, and mechanical fastening are three common joining methods used on polymer composites. Mechanical fastening and adhesive bonding are commonly used for joining large composite parts [28].

Ultrasonic welding (UW) is widely used for joining thermoplastic composites to similar and dissimilar materials. J. Tsujino et al. investigated the weld characteristics by welding polypropylene (PP) and polymethyl methacrylate (PMMA), respectively, with a frequency ranging from 27 kHz to 180 kHz. The authors found out that higher weld strength was obtained at higher frequencies (67 kHz to 180 kHz) than at lower frequencies (27 kHz to 40 kHz) because of an increase in vibration velocity [29]. W. Tao et al. investigated the effects of weld time (W_t_) and use of an energy director (ED) on the strength of carbon/polyether-ether-ketone (C/PEEK) joints using UW. Testing was performed with weld times ranging from 0.7 s to 1.1 s with a 0.1 s interval, and two configurations were welded: without an ED and with a flat polyether-ether-ketone (PEEK) ED of 0.45 mm thickness. I. F. Villegas et al. investigated ultrasonic welding of carbon/epoxy (CF/EP) and CF/PEEK using a 50 µm thick polyethylenimine (PEI) coupling layer (C/EP-PEI-C/PEEK). Welding EP to EP composites with PEI as coupling layer (C/EP-PEI-C/EP) composites was performed as a comparison. It was found that C/EP-PEI-C/PEEK had a larger unwelded area of 25% of the total overlap area as compared to C/EP-PEI-C/EP with only 5% unwelded area. It was explained that at the PEI-PEEK interface, PEEK needs to melt first. However, PEEK has a higher T_m_ than PEI, and PEI would soften and flow first. Even with a larger unwelded area, C/EP-PEI-C/PEEK yields higher lap shear strength (LSS), and it could be explained that the stiffness of C/EP and C/PEEK is different and that C/EP-PEI-C/EP requires two coupling layers (one on each interface), resulting in a thicker weld line. It was also observed from the EP-PEI interphase that the size of the epoxy sphere dispersed in PEI resin decreased towards the PEI layer [30].

F. Lionetto et al. investigated the effects of two different thicknesses (75 µm and 250 µm) of polyvinyl butyral (PVB) film as coupling layers for ultrasonic welding of CF/EP. The results indicated that using the 250 µm film yielded a slightly higher LSS of 27.9 MPa as compared to the 75 µm film with an LSS of 24.3 MPa. The failure happened at the fiber and polyvinyl butyral (PVB) interface, as PVB partially penetrated the EP during curing. This caused macro-mechanical interlocking between the EP composite and PVB film [31]. E. Tsiangou et al. also investigated ultrasonic welding of CF/PEI to CF/EP composites using PEI film as a coupling layer. Two thicknesses of the coupling layer were used (60 µm and 250 µm), and a combination of coupling layer and loose PEI film was also welded for comparison. The results indicated that using a coupling layer with a loose film produced the highest LSS of 37.7 MPa. Welding without PEI film yields lower LSS due to large unwelded areas and thermal damage to the coupling layer. Due to the coupling layer being fixed on the TS adherend, it presents more difficulty in following the contour of the thermoplastic interface during welding, and hence, there is less contact area. This results in less friction and, therefore, a lack in the flow of resin. When a thicker coupling layer (250 µm) was used, it was found that the LSS increased due to an increase in resin flow [21]. G. Gohel et al. carried out a study on ultrasonic welding of CF/EL to CF/EP composite using 0.2 mm EL film and EL powder with 2 different masses of 0.155 mg/mm^2^ and 0.31 mg/mm^2^ as the coupling layer. The joint with EL film showed the lowest LSS of 3.12 MPa, as it was completely removed from the epoxy adherend, indicating poor adhesion between the film and the EP adherend. The use of 0.155 mg/mm^2^ of EL powder yielded a higher LSS of 5.6 MPa, and increasing the mass to 0.32 mg/mm^2^ increased the LSS to 9.8 MPa. Increasing the amount of powder ensured that there was sufficient resin for improved flowability and melting characteristics [32].

L. Zweifel et al. investigated the interphase of epoxy composites with three different co-cured thermoplastic layers. The thickness of the thermoplastic film used was 125 mm. Clear interphase formation with a thickness of approximately 10–20 µm between PMMA and epoxy could be seen with a concentration gradient [20]. E. Tsiangou et al. welded different combinations of epoxy composite to PEEK composites with two different thickness of PEI coupling layer of 60 and 250 µm and two ED materials, PEI and PEEK. Epoxy spheres were observed in the PEI ED from the cross-section observed under SEM when using a thinner coupling layer of 60 µm. This indicated that the coupling layer flowed and was affected during welding. However, the coupling layer remained unaffected by welding when using a thicker coupling layer of 250 µm. The usage of a 250 µm coupling layer resulted in a fully welded area with a higher LSS of 40.8 MPa as compared to 34.9 MPa when using a 60 µm coupling layer [33].

Elium (EL) resin is a unique thermoplastic, recently developed by Arkema, that can be cured at room temperature and used for mass production in many industries such as automotive parts, energy, sporting goods, etc. [1,34,35,36,37,38,39,40,41,42,43,44,45,46,47,48,49]. Extensive research and testing on similar polymer welding materials, such as Elium composite ultrasonically welded to Elium composite, has been performed to investigate their mechanical properties [18,50,51]. However, ultrasonic welding of dissimilar polymer composites using Elium resin composite with epoxy (EP) resin composite had not been performed and was the focus of this research. The significance of this research is that it introduces the possibility of using Elium composite together with epoxy composite in many industrial applications.

## 2. Materials and Manufacturing

### 2.1. Materials

In the current research, two types of polymer composites were manufactured: thermoset composite and thermoplastic composite. Unidirectional (UD) dry carbon fibers of areal weight of 470 gsm manufactured by SAERTEX, Germany were used as the reinforcement system, and Elium^®^ 150 resin, provided by ARKEMA, France was used as the matrix system for thermoplastic composite [52]. Bisphenol A diglycidyl ether (DGEBA) based epoxy (AM-8937A) resin, supplied by WELLS ADVANCED MATERIALS (Shanghai, China) Co. Ltd. was used as the matrix system for thermoset composite. Elium is an acrylic-based resin, that can be cured at room temperature. Benzoyl peroxide initiator was mixed with Elium 150 with a 100:3 mixing weight ratio of resin to hardener to form high molecular weight acrylic co-polymer through radial polymerization [53]. Polyamine mixture-based epoxy (AM-8937B) hardener was mixed with resin with a 100:35 mixing ratio of resin to hardener to form the cross-linking [32].

#### Energy Director (ED) and Coupling Layer

**Figure 1:**
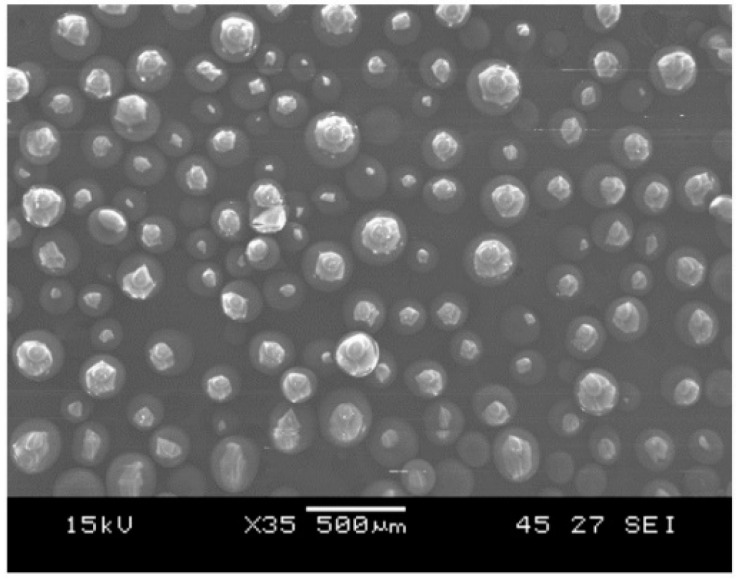
SEM image of PMMA particles.

Virgin PMMA film (A300-100) and mid toughened PMMA film (A300-103), provided by ARKEMA, were used as the loose flat ED in the current research work. They were cut to the size of 25.4 × 25.4 mm^2^, which was similar in size to the laminate welded area. The difference between the films is that the mid toughened film has rubber particles added as filler to strengthen the film. In order to weld the thermoset composite laminate to thermoplastic laminate, the thermoplastic particles were added into the thermoset adherend to make it weldable by forming a coupling layer. PMMA particles, Altuglass with particle size ranging from 150 to 200 µm, was procured from ARKEMA, United States and was used to form the coupling layer on the epoxy adherend to aid in the bonding. The PMMA particles as observed with SEM are shown in Figure 1.

### 2.2. Manufacturing of Composites

**Figure 2:**
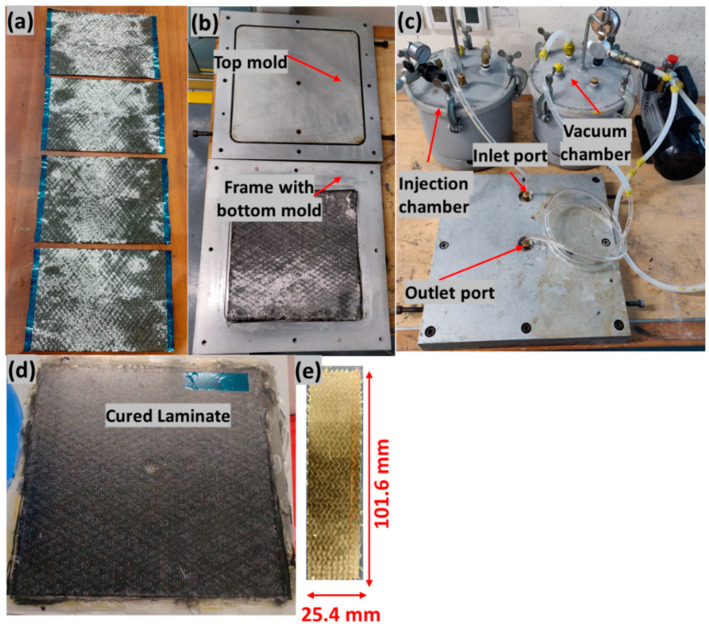
(**a**) Cut dry carbon fiber, (**b**) fiber in mold, (**c**) RTM setup, (**d**) cured laminate, (**e**) sample cut out from laminate.

Three types of composite laminates were manufactured: Elium composite, epoxy composite, and epoxy composite with coupling layer laminates. All of the composite laminates were manufactured using UD carbon fiber reinforcement, and the resin transfer molding (RTM) manufacturing process was used as shown in Figure 2. The mold consisted of three parts: top part, bottom part, and frame as shown in Figure 2b. The frame of 2 mm in thickness was used to control the thickness of the laminate. After mold preparation, four layers of UD dry carbon fibers were cut to the required dimensions of 270 × 270 mm^2^. The fibers were placed on top of each other in the bottom mold and at the center of the frame in alternating fiber directions of 0° and 90° (UD 0/90), as shown in Figure 2a.

The mold was then closed. Figure 2c depicts the RTM setup. The inlet port of the mold was connected to the pressure pot and the vacuum pot was connected on the other side, at the outlet port of the mold. The vacuum pump was used at the outlet to remove the entrapped air bubbles in the mold before injection.

To manufacture thermoplastic composite laminate, once the setup was ready, Elium 150 resin was prepared by mixing it with peroxide with a mixed ratio by weight of 100:3. The injection chamber was pressurized at 2 bar, and the resin was injected. The laminate was allowed to cure at room temperature for at least 1 h. After curing, the mold was post-cured in an oven at a temperature of 65 °C for 45 min, after which the mold was de-molded to remove the cured laminate. For manufacturing of epoxy composite laminate, after closing the mold and preparing the RTM setup, the mold was placed into the heat press and heated to a temperature of 50 °C. The resin was also prepared by mixing it with hardener in a 100:30 weight ratio at 50 °C. The resin injection was carried out at 50 °C. Once the resin flowed out through the outlet and after closing both the inlet and outlet, the mold temperature was increased to 100 °C and was allowed to cure at this temperature for 5–8 min. Following this, the mold was cooled down to room temperature, and then the laminate was demolded. Figure 2d shows the cured laminate.

**Figure 3:**
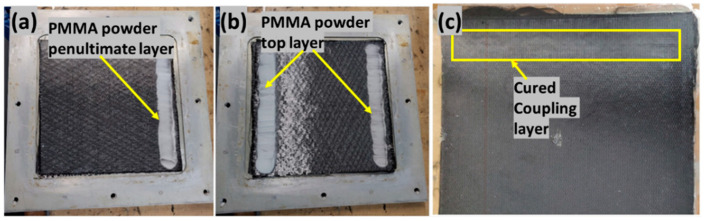
Powder applied on (**a**) penultimate layer, (**b**) outer adherend, (**c**) EP laminate with coupling layer.

To manufacture epoxy composite laminate with PMMA powder as coupling layer, the powder was sprinkled on the top layer in one configuration and on both the top and penultimate layer (fourth layer and third layer) in the other configuration. The fibers were placed in the mold as shown in Figure 3. It should be noted that the powder was sprinkled only on the intended weld area and following the fiber direction. The mold was closed, heated to 180 °C and kept at that temperature for 5–8 min to melt the powder. The mold was then cooled to 50 °C for injection following a similar cycle of epoxy laminate manufacturing. Figure 3c shows the cured laminate with coupling layer. The coupling layer was observed to have a slightly different reflection as compared to another surface. The cured laminates were cut using a waterjet cutter into samples with dimensions of 25.4 mm × 101.6 mm, with the top layer fiber direction in the longitudinal direction in accordance with the ASTM D5868-01 standard for lap shear testing (refer Figure 2e). The manufactured composite laminates had a final thickness of 2 mm with a fiber volume fraction of 54%.

## 3. Experimental Methods

### 3.1. Ultrasonic Welding

**Figure 4:**
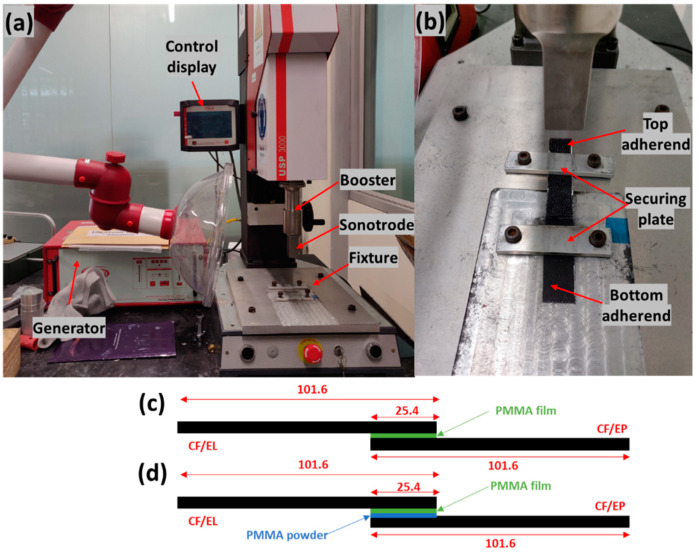
(**a**) Ultrasonic welding machine. (**b**) Close-up of the fixture welding configuration: (**c**) EL-EP, CF/EL composite with CF/EP composite, (**d**) EL-EP_PMMA, CF/EL composite with CF/EP composite with PMMA powder as a coupling layer.

The ultrasonic welding machine used in this research was acquired from ROOP TELSONIC ULTRASONIX LIMITED, Gandhinagar, Gujarat, India as shown in Figure 4a. It has a maximum output power of 3000 W, generates a frequency of 20 kHz, and is controlled by the AWC-6 microprocessor controller. In this research work, welded joints of CF/EL to CF/EL and CF/EL to CF/EP were investigated by varying the weld pressure and weld time, and hence, a constant time mode was used. The amplitude was kept at a constant of 37.5 µm (50%) [54] and hold time of 3s. The fixture was designed to weld the lap shear samples as shown in Figure 4b.

The three welding configurations investigated in the current research work are shown in Figure 4c,d. All configurations were welded with a flat ED using three layers of PMMA film with a total thickness of 0.45 mm (0.15 mm thick per layer). The samples were placed on the fixture with the PMMA film in between, as shown in Figure 4. The sonotrode was moved down at a pressure of 0.15 bar to hold and align the weld area to the surface of the sonotrode. After the sample was aligned, it was secured by tightening the screws of the plates.

### 3.2. Lap Shear Testing

**Figure 5:**
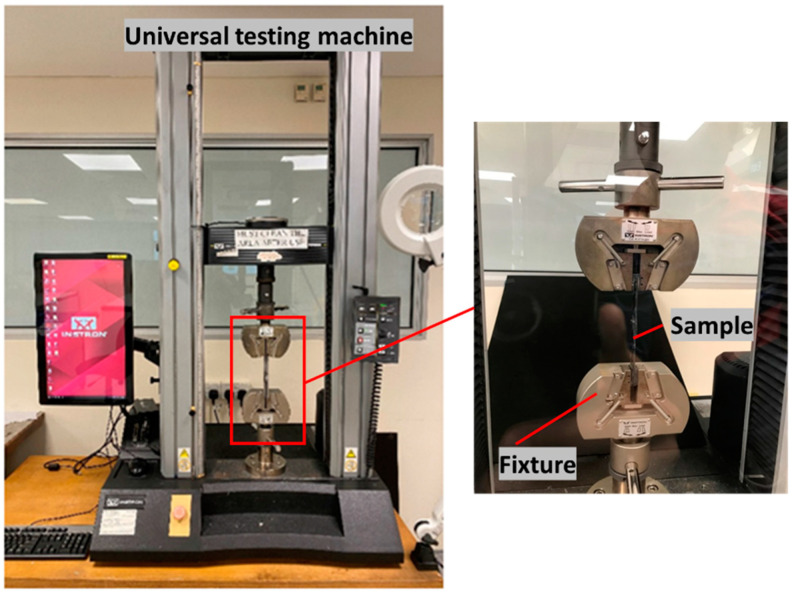
Universal testing machine used for static lap shear testing.

Lap shear testing was carried out to obtain the static lap shear strength of the weld joints for easy usage and good reproducibility [55]. The testing followed the ASTM D5868-01 standard [56]. A universal testing machine, INSTRON 5569, was used for the testing (refer to Figure 5). The test was conducted at a constant speed of 13 mm/min. A load-displacement curve was plotted during the testing. The joints were welded by overlapping of the sample, and hence, the tabs of similar thickness were bonded to each adherend using cyanoacrylate super glue to offset the thickness so that the sample could be clamped on the machine. Load-displacement curves were plotted, and the maximum LSS was obtained by dividing the peak load and the welded area. For each configuration, at least three samples were tested. An optical microscope (OLYMPUS S Z X7) was used to check the interface of the welded area. The welded samples were cut in cross-section to observe the interface. Weld line, weld thickness, the interaction between layers, and failure mechanisms were analyzed using a microscope. Scanning Electron Microscope (SEM) (JEOL SEM 5 600 LV) was used for the fractography investigation to observe the failure mechanisms of the top and bottom surfaces of the laminates. It was also used to observe the interface of the cross-section of the welded joint to study the interphase between different layers.

### 3.3. Initial Trials

#### Elium Composite to Epoxy Composite

Welding of Elium to epoxy composite without a coupling layer using 103 grade films was performed using different welding parameters to check the maximum lap shear strength. However, as the thermoset adherend had the inherent property of not being able to melt at higher temperature, a very poor bond was formed at the interface. A maximum LSS of 2.95 MPa was achieved using 103 grade films, with welding conditions of weld pressure (W_p_) of 3 bar and weld time (W_t_) of 2 s. As direct welding of the thermoset adherend to thermoplastic adherend could not be performed, the alternate approach was to form a coupling layer of the thermoplastic at the welding interface of the thermoset composite adherend. In the current research, the coupling layer on the carbon/epoxy adherend was created with varying masses of PMMA powder sprinkled on the layers. The configuration of the location and sprinkled mass is shown in Table 1. It should be noted that the indicated mass was in grams per welded area and the welded area was 25.4 × 25.4 mm^2^.

**Figure 6:**
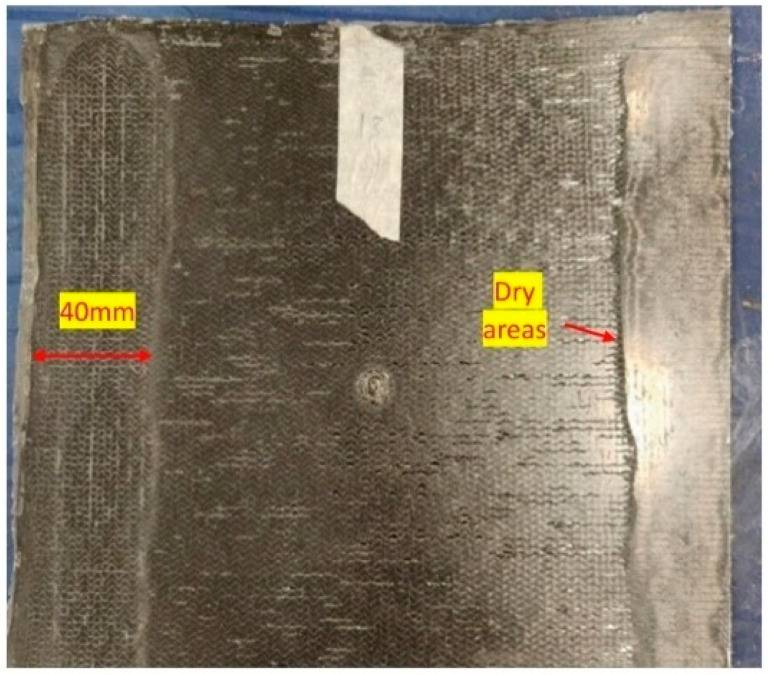
Defects of laminate with excessive powder.

In the manufactured samples of EL-EP_0.5_0.5 and EL-EP_1, dry spots were observed on the laminate, as shown in Figure 6. This was because of the excessive amount of sprinkled PMMA powder, which resulted in excessive compression of the fibers. This resulted in a decrease in the permeability of the fiber, making it difficult for the resin to flow into the fabric preform [57,58]. It was noticed that the area where the powder was excessively spread, once melted, could not be further compressed. The thickness of the area was 2.3 mm, which was too thick; hence, these two configurations were not investigated for welding trials. A full factorial design was used in this research [59]. Based on the initial trials, the ranges of W_t_ and W_p_ were selected, as shown in Table 2.

## 4. Results and Discussion

Welding and testing of different configurations with different parameters were carried out to optimize the LSS. Three weld configurations for Elium composite welded to epoxy composite with a coupling layer were optimized, as shown in Table 2. Failure surfaces and cross-sections of the interfaces were studied using an optical microscope and SEM to understand the failure mechanism and interphase of the different matrices. In this section, the results obtained are shown and discussed.

### 4.1. Lap Shear Testing of EL-EP Composites with Coupling Layer

Three samples were tested for each configuration. Herein, the load-displacement curves were the best representative curves from the trials that were closest to the average LSS value. Similar curves could be seen in the load-displacement curves of the individual configurations. During testing, there was a linear increase in load until it reached the maximum value, and a sharp drop in load was observed, signifying complete failure of the sample. The maximum load for each configuration could be obtained from the load-displacement curve. The maximum lap shear stress was calculated based on the maximum obtained load divided by the welded area. As seen from the load-displacement curves and LSS vs. weld time graph, at constant pressure, when the weld time increased, the LSS also increased. The maximum LSS was reached when increasing up to a certain weld time. A further increase in weld time led to a decrease in LSS. The same was true for the sample with optimized weld time that was capable of withstanding loads with the largest displacement. This trend was observed in a study by S.K. Bhudolia et al., where CF/EL was ultrasonically welded with CF/EL using flat and integrated ED by varying the weld time [51]. It is explained using the relationship of energy at the interface with the weld time. Better melting and flowing of the resin can be achieved with higher energy at the interface. At the lower time, there is not enough energy to melt the film and form a good bond with the adherends. When the optimal time is reached, both the film and the resin of the adherend are melted and form the best bond due to fusion bonding. When the time is increased further, excessive energy causes excessive melting and flowing of resin. The resin is squeezed out of the interface, and damage on the adherend can be observed [51].

**Figure 7:**
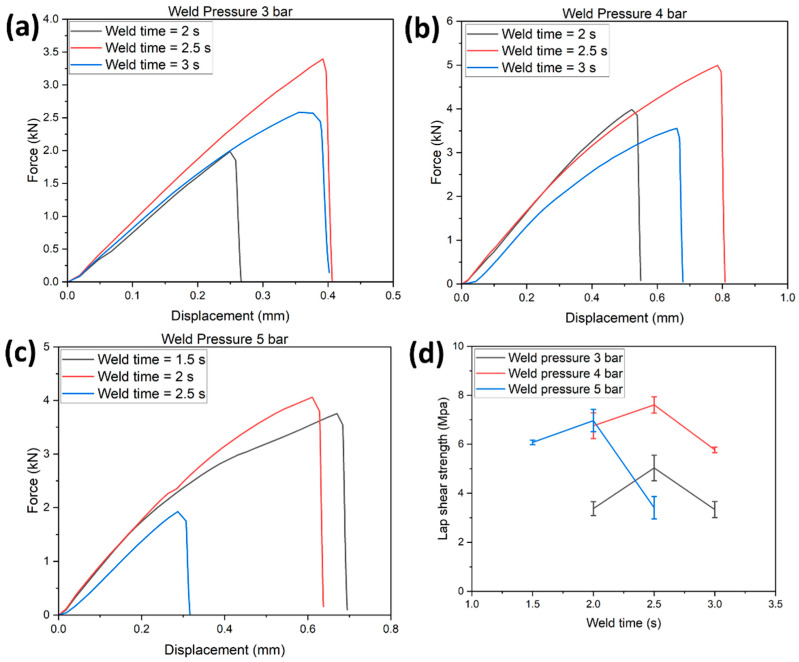
Load-displacement curve for EL-EP (Elium^®^-Epoxy)_0.125_0.125: (**a**) Weld pressure = 3 bar, (**b**) Weld pressure = 4 bar, (**c**) Weld pressure = 5 bar. (**d**) Lap shear strength vs. Weld time.

Figure 7a–c represents the load-displacement curve of the EL-EP_0.125_0.125 tested samples at weld pressures of 3, 4, and 5 bar and weld times of 2, 2.5, and 3 s. At a specific pressure, an increase in weld time increased the maximum load and displacement until the specific weld time where further increases would decrease the maximum load and displacement. Figure 7d shows the LSS vs. weld time graph of EL-EP_0.125_0.125 tested samples at weld pressures of 3, 4, and 5 bar. The maximum LSS using weld pressures of 4 and 5 bar were similar and much lower at 3 bar, indicating that higher pressure is required. When the pressure increased from 4 to 5 bar, the optimal weld time required decreased from 2.5 to 2 s. The maximum LSS was found to be 7.61 MPa in samples welded at 4 bar and 2.5 s, and the minimum LSS was 3.34 MPa in samples welded at 3 bar and 3 s.

**Figure 8:**
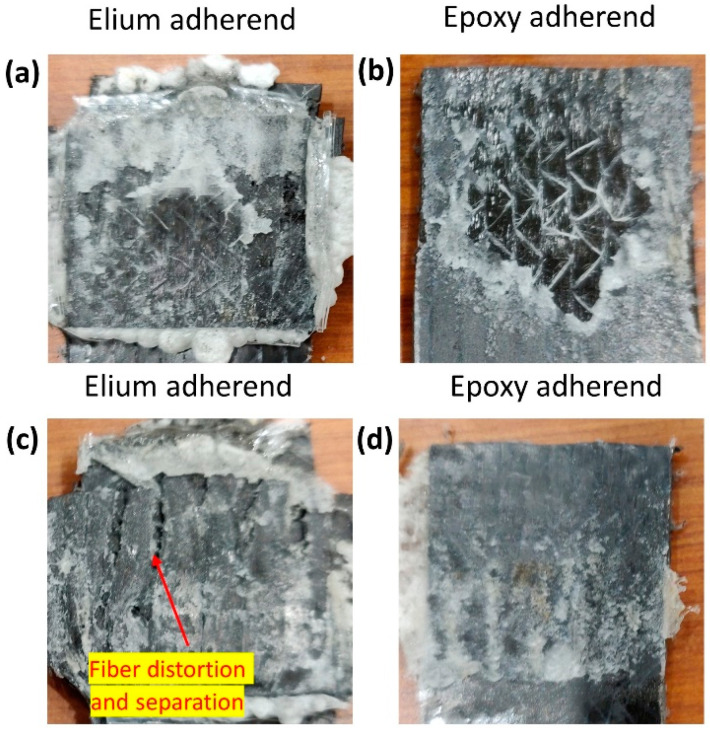
Fracture surfaces of EL-EP(Elium^®^-Epoxy)_0.125_0.125. (**a**,**b**) Maximum Lap shear strength and (**c**,**d**) minimum Lap shear strength.

Figure 8 shows the fracture surface of EL-EP_0.125_0.125 of both the Elium and epoxy adherend that yielded the maximum and minimum LSS. In Figure 8a, the film can be seen to be melted and bonded to the adherend. There is also some resin from the coupling layer that can be seen bonded on the adherend. Similarly, the film is also melted in the Elium adherend, as seen in Figure 8c. However, fiber distortion and separation were observed, and this was due to a higher weld time of 3 s. A clearer understanding can be obtained from the epoxy adherend. From Figure 8b, the coupling layer was melted, and a larger area was bonded to the melted film, signifying partial cohesive failure, whereas in Figure 8d, there was no melting of the coupling layer and hence, no bonding between the two adherends.

**Figure 9:**
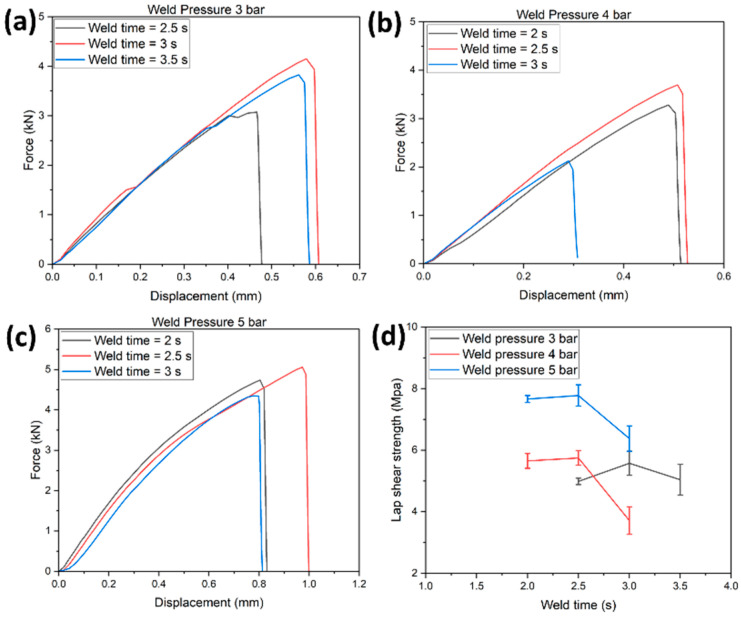
Load-displacement curve for EL-EP(Elium^®^-Epoxy)_0.25: (**a**) Weld pressure = 3 bar, (**b**) Weld pressure = 4 bar, (**c**) Weld pressure = 5 bar. (**d**) Lap shear strength vs. weld time.

Figure 9a–c represents the load-displacement curve of EL-EP_0.25 tested samples at weld pressures of 3, 4, and 5 bar and weld times of 2, 2.5, 3, and 3.5 s. A similar trend can be observed at a specific pressure; an increase in weld time increases the maximum load and displacement until the specific weld time where further increase would decrease maximum load and displacement. Figure 9d shows the LSS vs. weld time graph of EL-EP_0.25 tested samples at weld pressures of 3, 4, and 5 bar. The maximum LSS using weld pressures of 3 and 4 bar was similar and increased at 5 bar, indicating that higher pressure is required. When the pressure increased from 3 to 4 and 5 bar, the optimal weld time required decreased from 3 to 2.5 s. The maximum LSS was found to be 7.78 MPa in samples welded at 5 bar for 2.5 s and the minimum LSS was 3.71 MPa in samples welded at 4 bar for 3 s.

**Figure 10:**
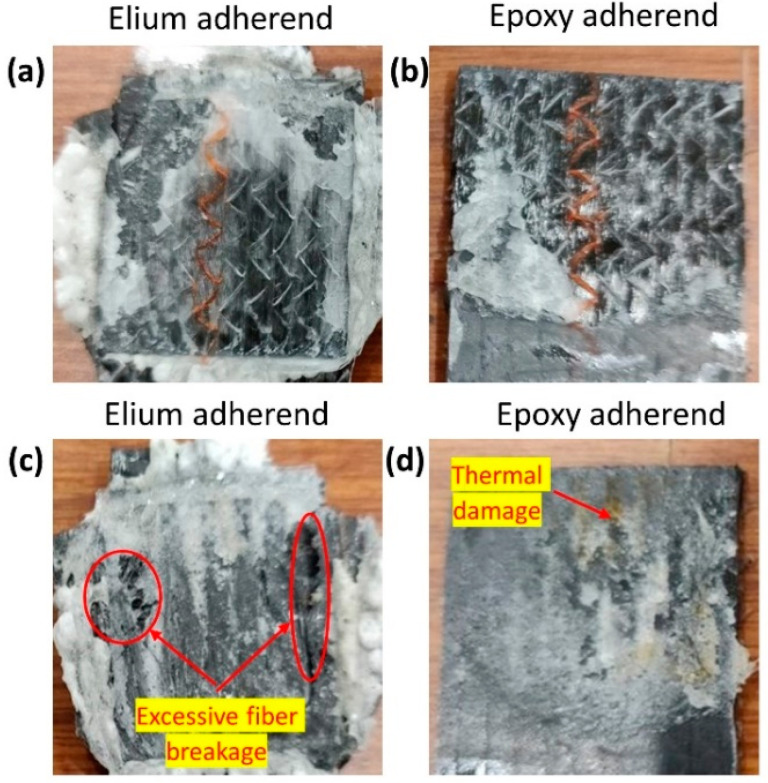
Fracture surfaces of EL-EP(Elium^®^-Epoxy)_0.25 at (**a**,**b**) maximum Lap shear strength and (**c**,**d**) minimum Lap shear strength.

Figure 10 shows the fracture surface of EL-EP_0.25 of both the Elium and epoxy adherend that yielded the maximum and minimum LSS. From Figure 10a, it can be seen that the film melted and bonded to the adherend. Some resin from the coupling layer was also seen to be bonded to the adherend. The film was also melted in the Elium adherend, as seen in Figure 10c. However, excessive fiber breakage could be seen especially at the side of the adherend due to the higher weld time of 3 s. Figure 10b shows that most of the coupling layer was bonded to the Elium adherend, exposing the bare fibers. This indicates that there was an adhesive failure between the coupling layer and fibers. In Figure 10d, there is no melting of the coupling layer, and some thermal damage can be seen from the brown areas due to a higher weld time of 3 s. Hence, there was no bonding for the minimum LSS condition.

**Figure 11:**
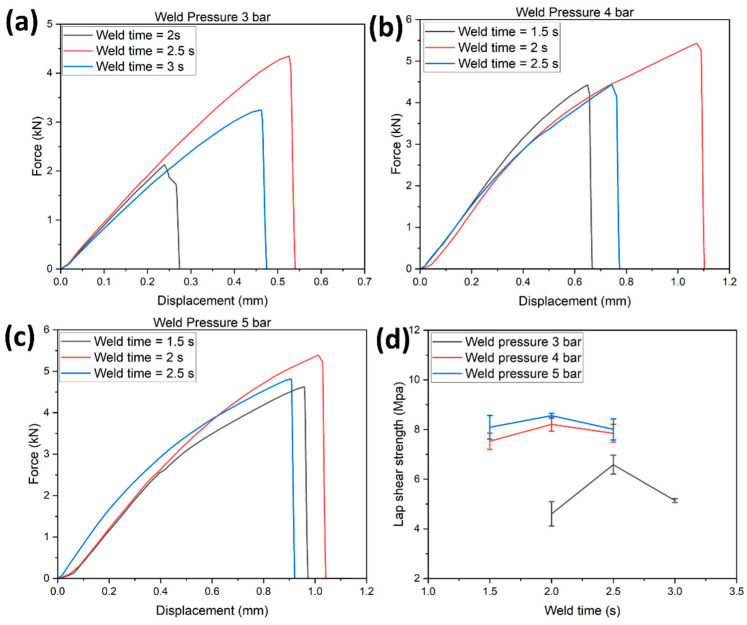
Load-displacement curve for EL-EP(Elium^®^-Epoxy)_0.25_0.25: (**a**) Weld pressure = 3 bar, (**b**) Weld pressure = 4 bar, (**c**) Weld pressure = 5 bar. (**d**) Lap shear strength vs. weld time.

Figure 11a–c shows the load-displacement curve of tested EL-EP_0.25_0.25 samples at weld pressures of 3, 4, and 5 bar and weld times of 1.5, 2, 2.5, and 3 s. A similar trend could be observed: at a specific pressure, an increase in weld time increased the maximum load and displacement until the specific weld time where a further increase would decrease the maximum load and displacement. Figure 11d shows the LSS vs. weld time graph of tested EL-EP_0.25_0.25 samples at weld pressures of 3, 4, and 5 bar. The maximum LSS using a weld pressure of 4 and 5 bar was similar and much lower at 3 bar, indicating that higher pressure was required for the resin to penetrate the fiber. When the pressure increased from 4 to 5 bar, the optimal weld time required decreased from 2.5 to 2 s. This trend was the same as the test result for EL-EP_0.125_0.125. The maximum LSS was found to be 8.56 MPa in samples welded at 5 bar for 2 s and the minimum LSS was found to be 4.61 MPa in samples welded at 3 bar for 2 s.

**Figure 12:**
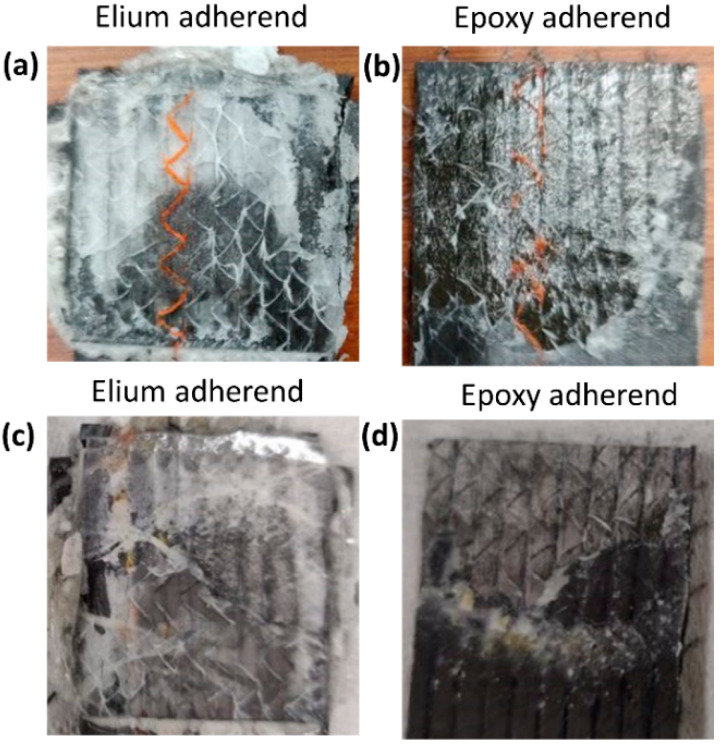
Fracture surfaces of EL-EP(Elium^®^-Epoxy)_0.25_0.25 (**a**,**b**) maximum LSS and (**c**,**d**) minimum LSS.

Figure 12 shows the fracture surface of EL-EP_0.25_0.25 of both the Elium and epoxy adherend that yielded the maximum and minimum LSS. A similar type of failure could be seen for the configuration with maximum LSS. From Figure 12a, the film can be seen to be melted and bonded to the adherend. Some resin from the coupling layer was also seen to be bonded on the adherend. The film was also melted in the Elium adherend, as seen in Figure 12c. Figure 12b shows that most of the coupling layer was detached and bonded to the Elium adherend, exposing the bare fibers. This indicates that there was a partial cohesive or near interfacial failure between the coupling layer and fibers. In Figure 12d, approximately half of the welded area of the coupling layer is un-melted, and the other half is debonded from the fiber. This could be due to insufficient contact force between the film and coupling layer due to the low pressure of 3 bar, hence resulting in an unevenly welded area.

**Figure 13:**
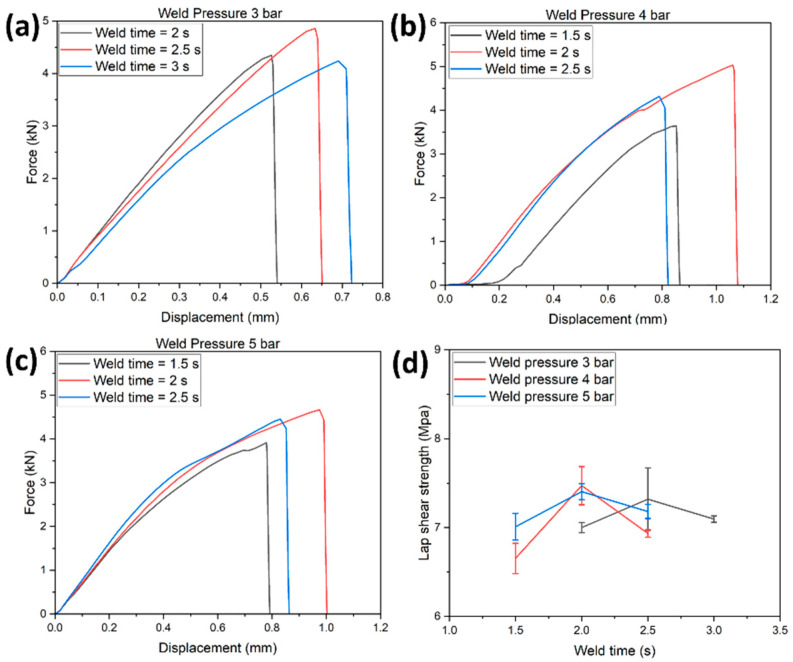
Load-displacement curve for EL-EP(Elium^®^-Epoxy)_0.5: (**a**) Weld pressure = 3 bar, (**b**) Weld pressure = 4 bar, (**c**) Weld pressure = 5 bar. (**d**) Lap shear strength vs. weld time.

Figure 13a–c represents the load-displacement curve of tested EL-EP_0.5 samples at weld pressures of 3, 4, and 5 bar and weld times of 1.5, 2, 2.5, and 3 s. A similar trend could be observed at a specific pressure: an increase in weld time increased the maximum load and displacement until the specific weld time where a further increase would decrease the maximum load and displacement. Figure 13d shows the LSS vs. weld time graph of tested EL-EP_0.5 samples at weld pressures of 3, 4, and 5 bar. The maximum LSS using weld pressures of 3, 4, and 5 bar were similar to each other, indicating that the pressure within this range was not a factor that would impact the LSS. When the pressure increased from 3 to 4 and 5 bar, the optimal weld time required decreased from 2.5 to 2 s. The maximum LSS was found to be 7.47 MPa in samples welded at 4 bar for 2 s and the minimum LSS was 6.65 MPa in samples welded at 4 bar for 1.5 s.

**Figure 14:**
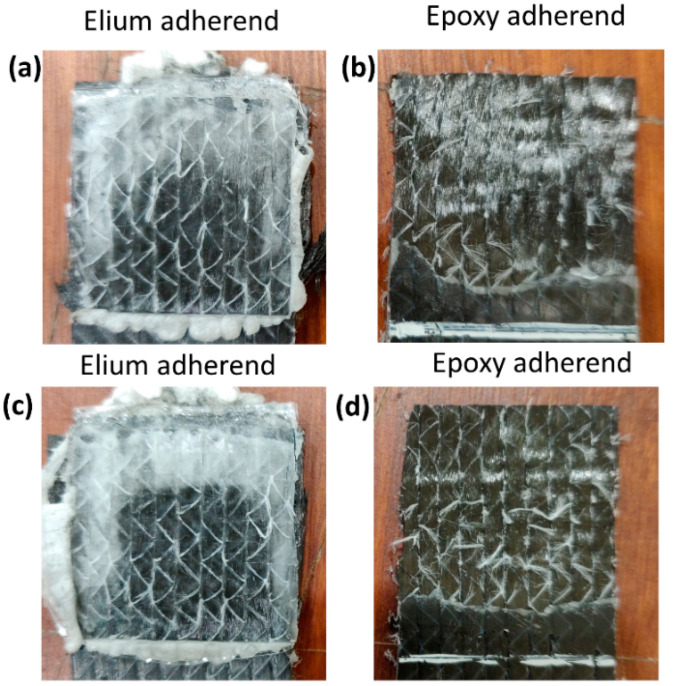
Fracture surfaces of EL-EP(Elium^®^-Epoxy)_0.5 (**a**,**b**) maximum Lap shear strength and (**c**,**d**) minimum Lap shear strength.

Figure 14 shows the fracture surfaces of EL-EP_0.5 of both the Elium and epoxy adherend that yielded the maximum and minimum LSS. The values of both maximum and minimum LSS were similar as compared to other configurations where the differences in LSS were large. Hence, similar failures could be seen from the two adherends. In both Figure 14a,c, the film is observed to be melted and bonded to the adherend, and chunks of coupling layer can also be seen. The coupling layer was detached from the fiber, exposing the bare fibers and indicating that adhesive failure occurred between the coupling layer and the fiber, as shown in Figure 14b,d.

**Figure 15:**
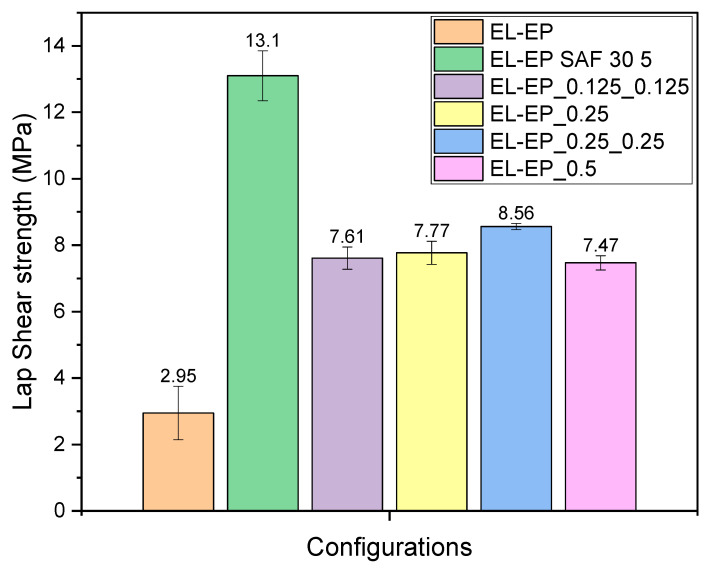
Maximum Lap shear strength comparison for each bonded configuration.

Figure 15 illustrates the maximum LSS achieved for all combinations of different configurations. The results obtained from welding Elium to epoxy composite served as a benchmark for the minimum achievable LSS. The LSS results obtained by G. Gohel et al. for welding of Elium to epoxy with 0.2 g/welded area of PMMA particle as coupling layer and semi-circular ED (EL-EP-ED) and adhesive bonding using SAF 30 5 (EL-EP SAF 30 5) were 9.83 MPa and 13.1 MPa, respectively [32]. These results were used as a benchmark for the upper limit. Table 3 lists the maximum LSS values tabulated for different weld configurations of Elium composite to epoxy composite. Among the weld configurations for Elium composite to epoxy composite with a coupling layer, EL-EP_0.25_0.25 showed the best result of 8.56 MPa. In comparison to welding without a coupling layer (EL-EP), the result from EL-EP_0.25_0.25 was higher by 190%, indicating the effectiveness of using a coupling layer. EL-EP_0.125_0.125 and EL-EP_0.25 had similar LSS values and percentage differences of 12% and 10%, respectively, compared to EL-EP_0.25_0.25. Even with a higher LSS, the weld time required was slightly lower, at 2 s compared to the other two configurations, which required a weld time of 2.5 s. EL-EP_0.5 and EL-EP-ELP yielded an even lower LSS and had a percentage difference of 15% and 24%, respectively, compared to EL-EP_0.25_0.25.

There was no significant change in LSS when comparing EL-EP_0.25 and EL-EP_0.25_0.25. This indicated that the penultimate layer had little to no effect on the bonding strength, as confirmed from the SEM images of the interface of these two configurations, which showed that the penultimate layer was unaffected after welding, as will be discussed later.

In comparison to EL-EP_0.25_0.25 with EL-EP-ED, where a different ED was used, EL-EP_0.25_0.25 showed a 13% lower LSS value. The results were expected, as using integrated/semi-circular ED proved to form better bonds than flat ED, based on the comparison performed by S.K. Bhudolia et al. [51]. However, integrated/semi-circular EDs do pose some difficulties in terms of the welding of complex geometries and proper control of injection parameters to form an ED without air bubbles during manufacturing [51]. Even though the LSS obtained using a flat ED was slightly lower, it was still useful in welding complex geometries such as tubular cross-sections.

Comparing ultrasonic welded and adhesive joints, EL-EP_0.25_0.25 showed an LSS value 35% lower than that from EL-EP SAF 30 5. Even though ultrasonically welded joints in this configuration are weaker in strength, there are other factors, such as manpower and time, where they are comparatively better than an adhesive joint. An adhesive joint requires time and effort for proper surface preparation before the application of adhesive. The welding time required is only 2 s as compared to 10 min for the adhesive to cure, and welding can significantly save the time required for bonding.

### 4.2. Microscopic Investigation of Cross-Sectional Interfaces of the Welded Adherends

The interfaces of EL-EP_0.25, EL-EP_0.25_0.25, and EL-EP_ELP with the maximum LSS were selected and investigated to understand the interphase between coupling layer and epoxy resin and the effect of coupling layer at the penultimate layer. The unwelded epoxy adherend of each configuration was also investigated and compared with the welded samples to understand if there were any changes to the interphase due to welding.

**Figure 16:**
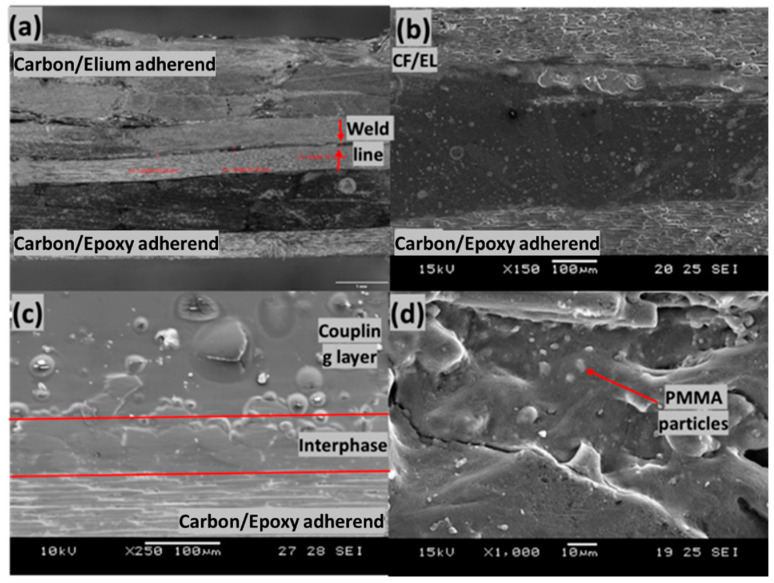
Cross-section of EL-EP(Elium^®^-Epoxy)_0.25. (**a**) Overall view, (**b**) Scanning Electron Microscope of welded interface, (**c**) top layer of unwelded epoxy adherend, (**d**) top layer of welded epoxy adherend.

Figure 16 shows the cross-section of the welded joint of the EL-EP_0.25 configuration that yielded the highest LSS. The total thickness of the ED and the coupling layer was approximately 850 µm, and it was reduced to a weld line thickness of 80.77 µm after welding, indicating that there was sufficient melting, flowing, and squeezing of resin. I. F. Villegas et al. observed the same phenomenon of reduction in the weld line due to the squeezing flow of melted film when welding epoxy composite to PEEK composite [30]. There was a larger decrease in thickness of 770 µm, as compared to EL-EP_0.25_0.25 with a decrease in thickness of 700 µm, and this could be caused by a higher welding time, resulting in more melting and flowing of resin.

We observed a deformation of the top adherend, as shown in Figure 16a, that might have been caused by the high weld pressure of 5 bar. Figure 16b shows the interface of the welded sample, and it can be seen that there was a decreasing concentration of PMMA particle from the epoxy adherend to the Elium adherend in the interphase. I. F. Villegas et al. observed a similar trend where epoxy spheres immersed in the PEI coupling layer decreased in concentration from the epoxy adherend to the PEEK adherend [30]. A similar trend can also be seen in Figure 16c, where melted and bonded PMMA particles were observed at the interphase and fewer particles were observed further above the interphase. Figure 16d shows the interphase above the epoxy adherend after welding, and good bonding between resin and the particles can be observed.

**Figure 17:**
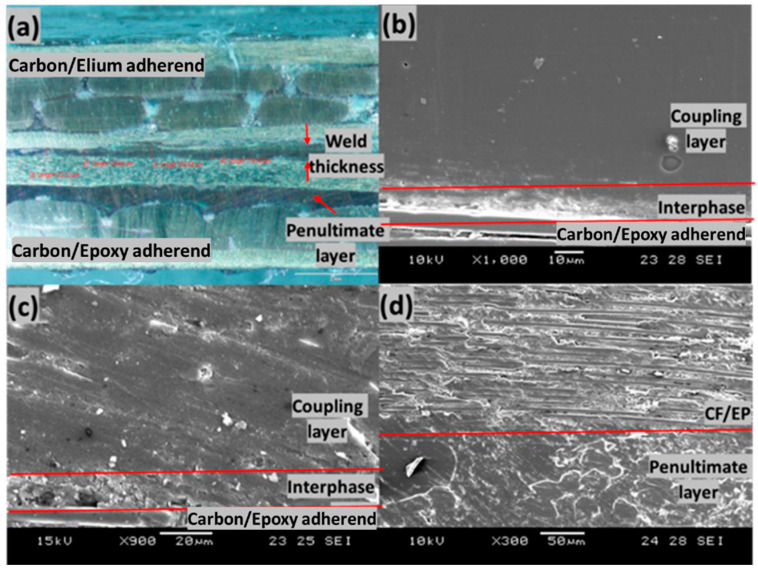
Cross-section of EL-EP(Elium^®^-Epoxy)_0.25_0.25. (**a**) Overall view, (**b**) top layer of unwelded epoxy adherend, (**c**) top layer of welded epoxy adherend, (**d**) penultimate layer of welded epoxy adherend.

Figure 17a shows the cross-section of the welded joint of the EL-EP_0.25_0.25 configuration, which yielded the highest LSS. The total thickness of the ED and the coupling layer was approximately 850 µm; it was reduced to a weld line thickness of 159.05 µm after welding, indicating that there was sufficient melting, flowing, and squeezing of resin. The total thickness of the ED and the coupling layer was the same as that in the EL-EP_0.25 configuration; however, the weld line thickness was slightly larger. This could be explained by the fact that EL-EP_0.25 was welded with a 0.5 s longer weld time, and hence, more resin was flowing and squeezing out from the sides, resulting in less resin at the interface. The interphase of melted PMMA particles and epoxy resin at the penultimate layer conformed to the surface of the fibers. The fibers at the penultimate layer and the layer below that were compressed, and this could be due to the manufacturing process where PMMA particles and fibers were compressed.

Figure 17b shows the interphase between the PMMA coupling layer and top carbon fibers of unwelded epoxy adherend. The appearance of the interphase had a rough texture and was a lighter shade of gray. The thickness was approximately 15 µm. The coupling layer on top of the interphase consisted of a pure PMMA matrix and had a darker shade of gray. This layer was very homogenous and smooth in texture. Figure 17c shows the interphase between the coupling layer and epoxy adherend after welding. The top of the interphase had a surface change from smooth to rough texture after welding, and it was similar to the texture of the interphase. The interphase itself remained unaffected by the welding and had a rough surface. E. Tsiangou et al. investigated the effect of coupling layer thickness using 60 µm and 250 µm films. When using a thinner coupling layer, the author observed that resin near the interphase flowed and changed the morphology of the interphase after welding. On the other hand, there was no effect on the interphase when using a thicker coupling layer, which could be explained by the fact that the interphase was protected, and flow was prevented [33]. The coupling layer formed from melted PMMA particles had a thickness of approximately 400 µm, and it was thick enough to protect the interphase from any changes. The PMMA particles on the penultimate layer were well melted and bonded together, as shown in Figure 17d.

### 4.3. Fractography of Failed Surfaces

The fracture surfaces of EL-EP_0.25, EL-EP_0.25_0.25, and EL-EP_ELP welded configurations were investigated in this section using SEM to understand the failure mechanism. The maximum LSS value of each configuration will be discussed first, followed by the minimum LSS value. The maximum LSS values for EL-EP_0.25 and EL-EP_0.25_0.25 were 7.78 MPa and 8.56 MPa, respectively. The minimum LSS values were 3.71 MPa and 4.61 MPa, respectively.

**Figure 18:**
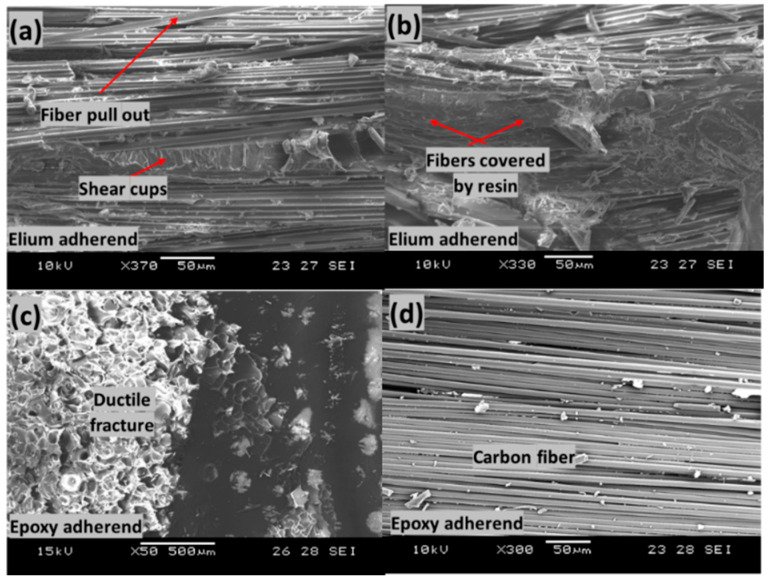
SEM fractography of EL-EP_0.25 with maximum LSS. (**a**,**b**) Elium adherend and (**c**,**d**) epoxy adherend.

Figure 18 shows the failed surfaces of the EL-EP_0.25 composite with an LSS value of 7.78 MPa when welded for 2.5 s at 5 bar. Figure 18a,b show fiber pull-out, shear cusps, and fibers covered by resin. Fiber pull-out and fibers covered by resin indicate a good bonding between the matrix and fiber. Shear cusps are formed due to interlaminar shear between the fiber and matrix when fibers are loaded during testing, which is also an indication of good bonding between fiber and matrix [49]. Therefore, there was plastic deformation at the PMMA film and interface between PMMA and Elium matrix. PMMA resin with ductile features could be seen on the epoxy adherend, indicating a ductile fracture as shown in Figure 18c [60,61]. Figure 18d seems to show bare carbon fibers, which can be easily misinterpreted as fibers without any resin forming a poor fiber/matrix bond. In some cases, resin is bonded to the fibers—for example, in an interlaminar shear failure. Due to the nature of overlap welding and tensile testing, interlaminar shear failure is expected at the interface. Defects such as debris due to abrasion between surfaces and shear cusps due to shear between fiber and matrix can be found [62]. Hence, there was a good fiber/matrix bond between the coupling layer and the fiber of epoxy adherend [63].

**Figure 19:**
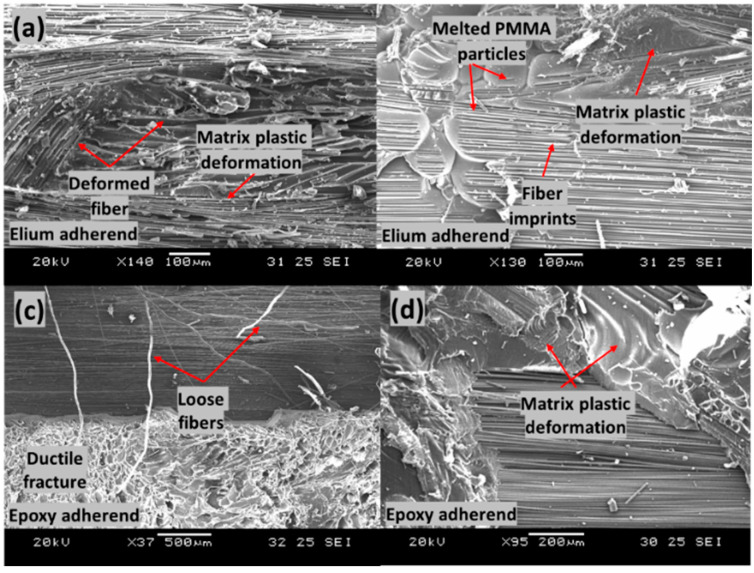
Scanning Electron Microscope fractography of EL-EP(Elium^®^-Epoxy)_0.25_0.25 with maximum LSS. (**a**,**b**) Elium adherend and (**c**,**d**) epoxy adherend.

Figure 19 shows the failed surfaces of EL-EP_0.25_0.25 composite with an LSS value of 8.56 MPa when welded for 2 s at 5 bar. Figure 19a shows good adhesion of the fiber with the matrix and the deformation of fiber together with matrix. Figure 19b shows a large area of melted PMMA particles bonded together on the Elium adherend. Fiber imprints could be seen everywhere, and this was due to the proper anchorage between the carbon fiber of the epoxy adherend and the coupling layer. Slow ductile fracture features and loose fibers can be seen in Figure 19c,d. A region with fibers similar to those in Figure 18d can be seen, indicating good bonding between fiber and coupling layer, and failure was due to interlaminar shear. Due to more plastic deformation of the bond between fiber and matrix, a slightly higher LSS was achieved as compared to that of EL-EP_0.25.

**Figure 20:**
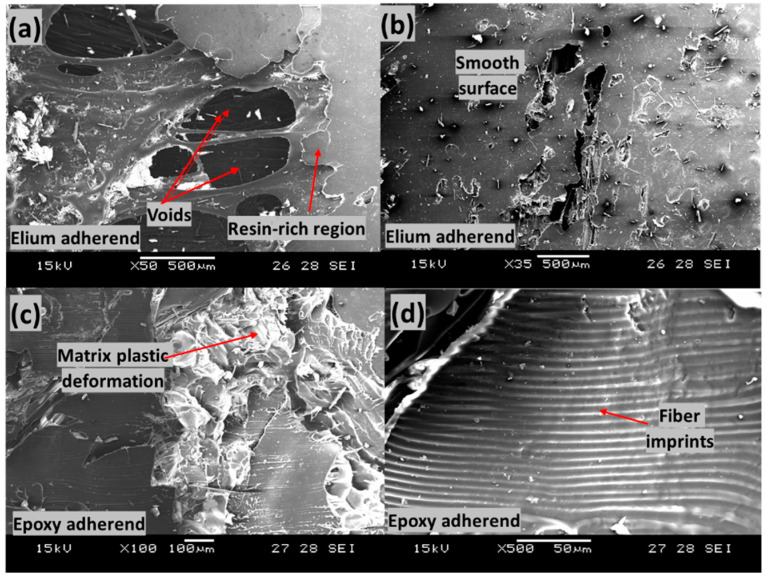
Scanning Electron Microscope fractography of EL-EP(Elium^®^-Epoxy)_0.25 with minimum LSS. (**a**,**b**) Elium adherend and (**c**,**d**) epoxy adherend.

Figure 20 shows the failed surfaces of the EL-EP_0.25 composite with an LSS value of 3.71 MPa when welded for 3 s at 4 bar. In Figure 20a, many broken fibers are observed on top of the fibers of Elium adherend and Elium matrix due to a slight increase in weld time, of 0.5 s. Many rich resin regions with smooth surfaces can be seen in Figure 20a,b. There was no plastic deformation, indicating that the resin did not bond well together. There are small areas of matrix plastic deformation shown in Figure 20c; however, most of the area contained smooth surfaces. It was also observed that on the smooth surfaces in Figure 20c, there were wavy textures, as shown in Figure 20d.

**Figure 21:**
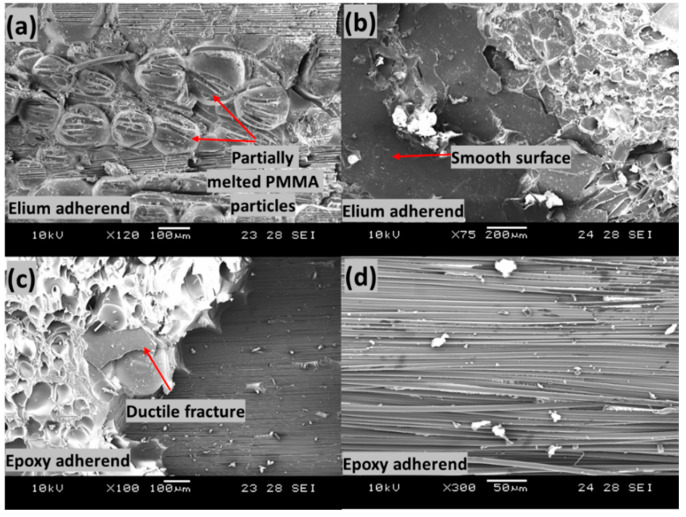
Scanning Electron Microscope fractography of EL-EP(Elium^®^-Epoxy)_0.25_0.25 with minimum LSS. (**a**,**b**) Elium adherend and (**c**,**d**) epoxy adherend.

Figure 21 shows the failed surfaces of the EL-EP_0.25_0.25 composite with an LSS value of 4.61 MPa when welded for 2 s at 3 bar. The same configuration with maximum LSS shown in Figure 21b had PMMA particles that were properly melted and bonded together, whereas in Figure 21a, the PMMA particles were partially melted and not well bonded together. A lower weld pressure of 3 bar was used, causing lower contact force, and hence, less compression and friction for melting and joining of PMMA particles. Areas of ductile fractures are observed from Figure 21c,d, with smooth areas indicating no bonding or fractures. Figure 21d shows the bare carbon of epoxy adherend, indicating poor bonding between the fibers and resin.

## 5. Conclusions

The main objective of the current research was to evaluate the weld characteristics of Elium composite ultrasonically welded to epoxy composite. Furthermore, an attempt to develop a manufacturing method where a coupling layer using thermoplastic particles was formed on the epoxy composite to strengthen the bond through the formation of interphase was successfully achieved. An experimental investigation was carried out to understand the effects of different parameters such as weld pressure, weld time, material, mass, and coupling layer location on weld strength. Afterward, static lap shear tests were performed on the welded samples, followed by studying the interphase formation of cross-sectional interfaces and the failure mechanisms of failed surfaces using microscopic and fractographic investigations. Following are the important findings from the current research:

- The EL-EP_0.25_0.25 configuration yielded the highest maximum LSS of 8.56 MPa when welded with a weld time of 2 s and weld pressure of 5 bar. The fracture surfaces indicated that the failure mode was a partial cohesive/near interfacial failure, as there were areas of failure within the resin and interfacial failure between the coupling layer and carbon fiber due to shearing.
- The EL-EP_0.125_0.125, EL-EP_0.25, EL-EP_0.5, and EL-EP_ELP configurations yielded the maximum LSS values of 7.61 MPa, 7.78 MPa, 7.47 MPa, and 6.88 MPa, respectively.
- The LSS of EL-EP_0.25_0.25 was 190% higher than that of EL-EP, signifying the effectiveness of the coupling layer. However, the obtained LSS was lower by 13% and 35%, respectively, compared to those of EL-EP-ED and EL-EP SAF 30 5. The use of semi-circular EDs is known to yield higher weld strength. The time required for welding was 2 s, as compared to 10 min for adhesives to cure, showing the time effectiveness of ultrasonic welding. These results confirmed the feasibility of welding dissimilar polymer composites using a coupling layer and provided the possibility of selecting thermoplastic and thermoset composites for different parts that are ultrasonically welded to form a product.
- In SEM images, epoxy and PMMA/Elium matrix interphase were observed to have a rough surface and remained largely unaffected by welding. There was an interphase change further away from the interphase to a rougher texture. There was little to no effect from the penultimate layer on the weld strength, as no interphase change could be observed after welding.
- From the fractography investigations, shear cusps, matrix plastic deformation, fiber imprints, fiber pull-out, and good adhesion between matrix and fiber were observed as features of configurations with maximum LSS, whereas voids, poor adhesion at the matrix/fiber interface, fiber breakage, and smooth surfaces were observed as features of configurations with minimum LSS.

While the current investigation paves the way to successful welding of Elium to epoxy composites, more study is currently under way to further optimize and increase weld strength by changing the coupling layers, using different fillers, varying the filler contents, and optimizing the entire weld process.

## Figures and Tables

**Table 1:** Location and mass of sprinkled polymethyl methacrylate (PMMA) powder.

Code Name	Top Layer (Grams Per Welded Area)	Penultimate Layer (Grams Per Welded Area)
EL-EP_0.125_0.125	0.125	0.125
EL-EP_0.25	0.25	0
EL-EP_0.25_0.25	0.25	0.25
EL-EP_0.5	0.5	0
EL-EP_0.5_0.5	0.5	0.5
EL-EP_1	1	0

**Table 2:** Design of experiments using different configurations.

Configuration	Weld Time (s)	Weld Pressure (bar)
EL-EP_0.125_0.125	1.5, 2, 2.5	3, 4, 5
EL-EP_0.25	2, 2.5, 3, 3.5	3, 4, 5
EL-EP_0.25_0.25	1.5, 2, 2.5, 3	3, 4, 5
EL-EP_0.5	1.5, 2, 2.5, 3	3, 4, 5

**Table 3:** Maximum LSS of different weld configurations.

Type of Weld	LSS (MPa)	SD (MPa)	% Change with Respect to		
EL-EP	2.95	0.80			
EL-EP SAF 30 5	13.1	0.75	**EL-EP**	**EL-EP-ED**	**EL-EP SAF 30 5**
EL-EP_0.125_0.125	7.6096	0.53	158%	−23%	−42%
EL-EP_0.25	7.7751	0.44	164%	−21%	−41%
**EL-EP_0.25_0.25**	**8.5597**	**0.09**	**190%**	**−13%**	**−35%**
EL-EP_0.5	7.4718	0.81	153%	−24%	−43%

## Data Availability

The data presented in this study are available on request from the corresponding author.
